# Type 1 autoimmune pancreatitis

**DOI:** 10.1186/1750-1172-6-82

**Published:** 2011-12-07

**Authors:** Yoh Zen, Dimitrios P Bogdanos, Shigeyuki Kawa

**Affiliations:** 1 Institute of Liver Studies, King's College Hospital and King's College London School of Medicine, Denmark Hill, London SE5 9RS, UK; 2 Center for Health, Safety and Environmental Management, Shinshu University, 3-1-1 Asahi, Matsumoto 390-8621, Japan

**Keywords:** IgG4, diagnosis, IgG4-related disease, pathology, pathogenesis

## Abstract

Before the concept of autoimmune pancreatitis (AIP) was established, this form of pancreatitis had been recognized as lymphoplasmacytic sclerosing pancreatitis or non-alcoholic duct destructive chronic pancreatitis based on unique histological features. With the discovery in 2001 that serum IgG4 concentrations are specifically elevated in AIP patients, this emerging entity has been more widely accepted. Classical cases of AIP are now called type 1 as another distinct subtype (type 2 AIP) has been identified. Type 1 AIP, which accounts for 2% of chronic pancreatitis cases, predominantly affects adult males. Patients usually present with obstructive jaundice due to enlargement of the pancreatic head or thickening of the lower bile duct wall. Pancreatic cancer is the leading differential diagnosis for which serological, imaging, and histological examinations need to be considered. Serologically, an elevated level of IgG4 is the most sensitive and specific finding. Imaging features include irregular narrowing of the pancreatic duct, diffuse or focal enlargement of the pancreas, a peri-pancreatic capsule-like rim, and enhancement at the late phase of contrast-enhanced images. Biopsy or surgical specimens show diffuse lymphoplasmacytic infiltration containing many IgG4^+ ^plasma cells, storiform fibrosis, and obliterative phlebitis. A dramatic response to steroid therapy is another characteristic, and serological or radiological effects are normally identified within the first 2 or 3 weeks. Type 1 AIP is estimated as a pancreatic manifestation of systemic IgG4-related disease based on the fact that synchronous or metachronous lesions can develop in multiple organs (e.g. bile duct, salivary/lacrimal glands, retroperitoneum, artery, lung, and kidney) and those lesions are histologically identical irrespective of the organ of origin. Several potential autoantigens have been identified so far. A Th2-dominant immune reaction and the activation of regulatory T-cells are assumed to be involved in the underlying immune reaction. IgG4 antibodies have two unique biological functions, Fab-arm exchange and a rheumatoid factor-like activity, both of which may play immune-defensive roles. However, the exact role of IgG4 in this disease still remains to be clarified. It seems important to recognize this unique entity given that the disease is treatable with steroids.

## Background

The entity "autoimmune pancreatitis (AIP)" was first proposed by Yoshida et al [1] in 1995, who described a case of steroid-responsive pancreatitis. That report described a case of a diffuse enlargement of the pancreas and irregular narrowing of the pancreatic duct, serologically associated with hyper-γ-globulinemia and anti-nuclear antibody (ANA) positivity [1]. The presence of pancreatitis, features of autoimmune disease, and responsiveness to immunosupression led to the connotation of AIP [1]. The term of AIP has been since used by other groups, and is now accepted worldwide. However, the first evidence of features compatible with AIP was described by Sarles et al[2]. in 1961, who reported the case series of an unusual pancreatitis associated with obstructive jaundice and hyper-γ-globulinemia, suggestive of an underlying autoimmune process. This form of pancreatitis was recognized as lymphoplasmacytic sclerosing pancreatitis or non-alcoholic duct destructive chronic pancreatitis based on distinct histological features in the 1990s [3,4]. Another landmark paper was published in the New England Journal of Medicine in 2001, where Hamano et al [5]. reported that serum IgG4 levels are specifically elevated in Japanese patients with AIP. An increase of IgG4 levels in AIP cohorts has been also confirmed in Western and Eastern countries [6,7]. The discovery of hyper-IgG4 has strengthened the concept of AIP. In addition, clinical and histological reviews of AIP patients provided evidence that AIP can be classified into 2 types: IgG4-related and non-related [8,9].

IgG4 is not only a serological marker but is also histologically detectable. The demonstration of pancreatic infiltration by IgG4^+ ^plasma cells reported in 2002 [10] was followed by studies reporting similar sclerosing lesions in various organs [11,12]. Hence, a new systemic disease entity, "IgG4-related disease", is proposed. This is based on the fact that synchronous or metachronous lesions can develop in multiple organs and the lesions are histologically identical irrespective of the organ of origin [13,14]. IgG4-related AIP is considered a pancreatic manifestation of IgG4-related disease.

## Subtypes and histopathology of AIP

Recent papers have provided evidence that there are two subtypes of AIP with different clinical and histological characteristics [8,9,15,16]. The classical form is called type 1 AIP, which is associated with elevated serum IgG4 levels and tissue infiltration by IgG4^+ ^plasma cells [15,16]. Type 2 AIP, which is not related to IgG4, was identified based on the histological features of neutrophilic infiltration into the pancreatic duct epithelium (granulocytic epithelial lesion: GEL) [17,18]. Type 1 appears to be the most predominant form of AIP. Clinical and histological features of both subtypes are summarized in Table 1. A Japanese nationwide study revealed that the annual number of patients with type 1 AIP is 0.71 per 100,000, which accounts for 2% of patients with chronic pancreatitis [19]. The exact prevalence of type 2 AIP is still unknown but it is less common than that of type 1 AIP [9,15].

**Table 1:** Comparison of type 1 and type 2 autoimmune pancreatitis (AIP).

	Type 1AIP	Type 2 AIP
Age	Adult	Child and adult
Gender	Predominantly male	Almost equal
Serum IgG4 levels	Elevated	Normal
Histology		
Histological nomenclature	Lymphoplasmacytic sclerosing pancreatitis (LPSP)	Idiopathic duct-centric pancreatitis
IgG4^+ ^plasma cells	Many	Rare
Granulocytic epithelial lesion	Absent	Present
Relapse rate	High	Low
Extra-pancreatic lesions	IgG4-related disease as shown in Table 2	Inflammatory bowel disease

**Figure 1:**
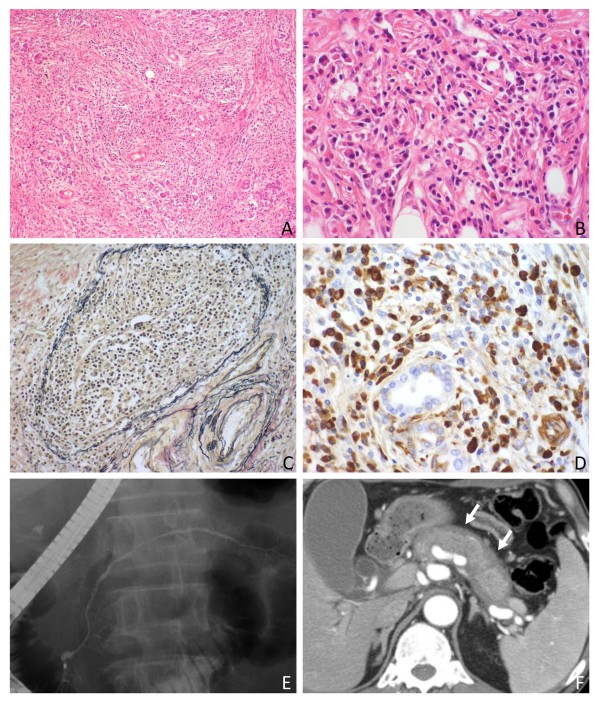
**Histological and radiological features of type 1 AIP**. A: Pancreas is inflamed with inflammatory cell infiltration and storiform fibrosis (H&E, ×100). B: Inflammatory cells consist of predominantly lymphocytes and plasma cells, and occasional eosinophils (H&E, ×400). C: A vein branch is obliterated by sclerosing inflammation (obliterative phlebitis) (Elastica van Gieson, ×200). D: Many IgG4+ plasma cells are identified (IgG4 immunostaining, ×400). E: ERCP shows diffuse irregularity of the pancreatic duct. F: The pancreas is diffusely enlarged with a peri-pancreatic rim (arrows) (contrast-enhanced CT).

Type 1 and type 2 AIP share some histological features including diffuse lymphoplasmacytic infiltration and fibrosis, but these are classifiable based on purely histological grounds [17,18,20]. The pathological synonym of type 1 AIP is lymphoplasmacytic sclerosing pancreatitis (LPSP) which is characterized by a predominantly lobular involvement with "storiform" fibrosis, obliterative phlebitis, and infiltration by many IgG4^+ ^plasma cells (Figure 1A-D) [3,20]. A diagnostic feature of type 2 AIP is GEL with or without lobular neutrophilic infiltration [17,18,20]. IgG4^+ ^plasma cells appear to be less conspicuous in type 2 AIP [21]. That is, the most important histological feature is IgG4^+ ^plasma cell infiltration in type 1 and neutrophilic epithelial injury in type 2 AIP. Given that the clinical features and pathogenesis of type 2 AIP are poorly defined, this review will only discuss type 1 AIP.

## Clinical features

Type 1 AIP predominantly affects adult males with > 90% of patients being more than 40 years of age. There is a male preponderance, with a male/female ratio of 3-4:1 [19,22,23]. The major symptom at onset is obstructive jaundice, which is caused by an enlargement of the pancreatic head or thickening of the bile duct wall [24-26]. Blood tests results include increased bilirubin and cholestatic enzymes, transaminasaemia, and occasionally elevation of carbohydrate antigen 19-9 (CA19-9) [24]. These findings mimic those found in pancreatic cancer, and indeed 2-3% of pancreatic head lesions surgically resected for suspected malignancy turned out to be type 1 AIP by histological examination [27,28]. Contrary to pancreatic cancer, the jaundice of type 1 AIP patients is quickly improved by steroid therapy or sometimes subsides spontaneously. Severe abdominal pain is a rare subjective symptom different from ordinary chronic pancreatitis, such as alcoholic pancreatitis [24]. In over half of cases, levels of pancreatic enzymes are mildly or moderately elevated [24]. Diabetes mellitus is a common complication, seen in about half of the patients, the majority showing type II diabetes. Steroid therapy can ameliorate glucose intolerance in some patients along with an improvement of pancreatitis, but may aggravate the diabetes in others, particularly older patients [29,30]. Diarrhoea due to impaired exocrine function is rare, but bentiromide or secretin testing shows somewhat impaired exocrine function [30]. This can be improved to some extent by steroid therapy [31]. Patients may have symptoms related to extra-pancreatic lesions such as lacrimal and salivary gland enlargement [13], cough or dyspnea caused by respiratory lesions [32], lumbago caused by hydronephrosis and retroperitoneal fibrosis [10], and polyuria caused by prostatic lesions [33-35].

## Serological characteristics

Elevated serum levels of IgG4 (> 135 mg/dL) are seen in more than 90% of patients [5,24]. This is the most sensitive and specific diagnostic test for type 1 AIP, with 95% sensitivity, 97% specificity, and 97% accuracy for discrimination from pancreatic cancer [5]. It is also useful for determining disease activity and predicting relapse [36-38]. Other commonly detectable immunological features include elevation of IgG (70%), γ-globulin (60%), and IgE (33%) [39,40]. Rheumatoid factor and ANA are also detectable in 30 to 40% of patients [39,40]. However, disease-specific autoantibodies such as anti SS-A/Ro and SS-B/La or anti-mitochondrial antibodies are extremely uncommon [24]. These findings suggest that patients with type 1 AIP can produce various autoantibodies but these antibodies lack disease specificity.

Complement C3 and C4 are reduced in 36% of patients, particularly those with high levels of circulating immune complexes, suggesting immune-mediated complement activation [41]. The complement activation system consists of classical, alternative, and mannose-binding lectin pathways. Given that the latter two seem less activated in type 1 AIP, the classical pathway may be predominantly operating [41].

The exact role of IgG4 in IgG4-related disease remains elusive. IgG4-type pathogenic autoantibodies can induce tissue damage, as in the case of anti-desmoglein 3 autoantibody in pemphigus vulgaris [42]. Few reports regarded IgG4-type autoantibodies as pathogenic in type 1 AIP. IgG4 was recently discovered to have two outstanding characteristics, Fab-arm exchange [43] and rheumatoid factor-like activity [44], both of which may play immune-defensive roles by eliminating IgG4-bound immune complex from the circulation. Fab-arm exchange results in bispecific antigen binding, which interferes with the formation of IgG4-associated immune complexes [44]. It was also demonstrated that IgG4 can bind to another IgG of any subclass via Fc-Fc interaction, and this IgG4 Fc-Fc interaction may represent an intermediate state of the Fab-arm exchange reaction [44-47], although the role of this function remains unclear. Further studies are needed to clarify whether IgG4 has beneficial or detrimental effects in IgG4-related disease including type 1 AIP.

## Radiological features

An important radiological feature is irregular narrowing of the pancreatic duct which can be detected by endoscopic retrograde cholangiopancreatography (ERCP) or magnetic resonance cholangiopancreatography (MRCP) (Figure 1E) [48,49]. The former is more sensitive but the latter is more widely used for diagnosis given its less invasive nature. Complete obliteration of the main pancreatic duct with dilatation of the distal duct, which is commonly found in patients with pancreatic cancer, is atypical in type 1 AIP.

Characteristic features of ultrasonography (US), computed tomography (CT) and magnetic resonance imaging (MRI) include diffuse or focal pancreatic enlargement, a peri-pancreatic capsule-like rim, enhancement at the late phase of contrast-enhanced images, and abnormal signal intensity on MRI [50-52]. Diffuse enlargement of the pancreas with loss of the cobble-stone architecture of the pancreatic surface is a common finding of type 1 AIP in all imaging modalities [50-52]. US characteristically shows diffuse low-echoic change with high-echo spots which was originally called "sausage-like" swelling [53]. The enlarged pancreas is associated with a capsule-like, low-density rim which appears more evident on contrast-enhanced CT or MRI, particularly at the early phase (Figure 1F) [50-52]. This is a radiological finding representing a fibrosing process extending into peri-pancreatic adipose tissue [50]. In contrast to normal pancreatic parenchyma which shows enhancement at the early phase of contrast-enhanced images, type 1 AIP is characterized by enhancement at the late phase (a delayed enhancement) [50-52]. Signal intensity of type 1 AIP on T1-weighted MRI is decreased compared to non-affected pancreatic parenchyma, sometimes appearing even lower than that of the liver [50]. T2-weighted MRI scans show increased signal intensity. Rare radiological findings include multiple small nodules [54], stone formation [55], and cystic degeneration [56], the latter two being more common at the late stage.

It is also important to identify extra-pancreatic lesions, the presence of which is indicative of type 1 AIP and makes a diagnosis of pancreatic cancer less likely. F-18 fluorodeoxyglucose positron emission tomography (FDG-PET) is also useful for this purpose given that extra-pancreatic lesions are usually positive for FDG-PET the same as type 1 AIP [57-59]. Gallium-67 scintigraphy is another modality to detect extra-pancreatic lesions, accumulation at the pulmonary hilum being a characteristic finding for type 1 AIP patients [60]. In our experience, extra-pancreatic lesions are sometimes detected at unexpected sites by FDG-PET or systemic CT/MRI.

## Diagnosis

A number of diagnostic criteria for type 1 AIP have been proposed so far from Japan, South Korea, and the United States (HISORt criteria of the Mayo Clinic) [36,37,61-63]. Despite minor differences among sites, diagnostic criteria include imaging features, serological abnormalities, and histological findings. Patients with typical imaging features (diffuse pancreatic enlargement with an irregular/narrow pancreatic duct) and elevated serum IgG4 levels, with or without other non-specific autoimmune abnormalities (e.g., ANA or hyper-IgG), fulfill all criteria. In a situation in which serological features cannot be detected, histological evidence of LPSP (e.g., diffuse lymphoplasmacytic infiltration, storiform fibrosis, and obliterative phlebitis) needs to be confirmed by biopsies. Immunostaining of IgG4 is also useful with ≥ 10 IgG4^+ ^plasma cells per high-power field in biopsy samples being supportive of LPSP [64]. However, it should be noted that ≥ 10 IgG4^+ ^plasma cells can be rarely identified in biopsy specimens from pancreatic cancer [64].

There are still *pro*s and *cons *with steroid trials approved based on most diagnostic criteria except the Japanese ones [36,37,61-63]. Given that pancreatic swelling dramatically improves even after a few weeks of steroid therapy, steroid trials would be a useful diagnostic test. Immunosupression must only be considered under restricted conditions, given that pancreatic cancer is the most likely differential diagnosis of type 1 AIP.

Surgically resected specimens showing typical features of LPSP with numerous IgG4^+ ^plasma cells can be diagnosed as type 1 AIP based purely on histological grounds. This is particularly important for retrospective reviews of cases without sufficient serological and radiological data. It is questionable whether or not findings of a needle biopsy are sufficient for the diagnosis. If a diagnosis of type 1 AIP is histologically suggested, imaging and serological data should be reviewed. In our experience, histological findings of type 1 AIP in the absence of consistent radiological or serological features are extremely unlikely.

## Treatment

An administration of an oral steroid is a standard treatment for type 1 AIP [65,66]. Most patients respond well to immunosupression with significant improvement of radiological and serological abnormalities [29,31]. Spontaneous remission can also occur particularly in patients with inactive disease. Normally, patients are started on 30-40 mg, or 0.6 mg/kg, per day of prednisolone for 2-4 weeks with careful monitoring of clinical features, and serological and imaging findings [67,68]. Amelioration of the clinical features is usually observed within 2 weeks, and clinical remission is frequently seen within 4 weeks. Thereafter, the dosage is reduced (tapering by 5 mg/day every 2 weeks over 2-3 months, to 2.5-7.5 mg per day), and a maintenance dose is required for 6 months to 3 years [67].

Relapse can be defined as symptomatic or radiologically detectable disease recurrence in not only the pancreas but also extrapancreatic organs. Biochemical or serologic recurrence alone is not considered relapse. Given that most relapses are observed within the first 3 years, patients with active disease (e.g. extremely high levels of serum IgG4 or multiple organ lesions) require prolonged treatment [65-67]. Cessation of maintenance therapy can be considered after six months in those cases with inactive disease. If steroid therapy is ineffective, clinical and serological data need to be reviewed in order to definitively exclude pancreatic malignancy. Other therapies which have been reported to be effective for highly active or refractory type 1 AIP are steroid pulse therapy [69], immuno-suppression with azathioprine or 6-mercaptopurine [70], and rituximab administration [71].

Treatment by resection is not recommended for type 1 AIP. However, even in large pancreatic centres, the surgical approach is still unavoidable for minor cases, for which malignancy cannot be entirely excluded. Surgeries can be also considered for large pseudocysts, which are rarely associated with type 1 AIP [72,73]. Interestingly, one paper described that recurrent rate after surgery appears lower than that after steroid therapy [15].

## Extra-pancreatic lesions and IgG4-related disease

Type 1 AIP is commonly associated with the involvement of other organs, the incidence of which was reported to be approximately 50% to 70% [9,15]. Type 1 AIP is now considered as a pancreatic manifestation of systemic IgG4-related disease, and extra-pancreatic lesions similar to those seen in the pancreas, can develop in the absence of pancreatic abnormalities [74,75]. Extra-pancreatic lesions may be identified at the same time as type 1 AIP or may appear afterward particularly during tapering doses of steroid or after completion of steroid therapy. As mentioned, extra-pancreatic lesions can precede the episode of type 1 AIP. IgG4-related disease has been identified in various organs based on histological evidence of IgG4^+ ^plasma cell infiltration, steroid responsiveness, or high serum IgG4 concentrations. Most IgG4-related lesions share clinicopathological characteristics irrespective of the organ of origin, but several organ-specific features have been identified [76]. As shown in Table 2 a variety of organs can be involved in IgG4-related disease. Almost all lesions have probably been discovered, but the possibility of an IgG4-related disease always needs to be considered for an unusual lesion at any site if it appears during the follow up in patients with known IgG4-related disease.

**Table 2:** A list of major IgG4-related disease in various organs.

Organ	Manifestation
Pituitary gland	Hypophysitis
Meningis	Pachymeningitis
Lacrimal gland	Chronic sclerosing dacryoadenitis (Mikulicz's disease)
Salivary gland	Chronic sclerosing sialadenitis (Küttner's tumour)
Lymph node	Lymphadenopathy with 5 histological subtypes
	- Multicentric Castleman disease-like (type I)
	- Follicular hyperplasia (type II)
	- Interfollicular expansion (type III)
	- Progressive transformation of germinal centre (type IV)
	- Nodal inflammatory pseudotumor (type V)
Thyroid	Thyroiditis or hypothyroidism
Skin	Pseudolymphoma
Breast	Inflammatory pseudotumour or mastitis
Lung	Inflammatory pseudotumour
	Focal or diffuse interstitial pneumonia
	Inflammation in bronchovascular bundles.
Pleura	Nodular pleuritis
Stomach	Chronic gastritis or ulcer
Vater's ampulla	Pseudotumourous enlargement
Pancreas	Type 1 autoimmune pancreatitis
Bile duct	Sclerosing cholangitis
Gallbladder	Lymphoplasmacytic sclerosing cholecystitis
Liver	Inflammatory pseudotumour
	Chronic active hepatitis (autoimmune hepatitis)
Retroperitoneum	Retroperitoneal fibrosis
Aorta/artery	Periaortitis/periarteritis
	Inflammatory aneurysm
Kidney	Tubulointerstitial nephritis
Peripheral nerve	Perineural mass

## Pathogenesis

Several genetic susceptibility factors for type 1 AIP have been identified as summarized in Table 3[77-81]. Findings obtained in one ethnic group are not always confirmed in patients in other groups, which raises the possibility that genetic susceptibility factors may be different among ethnic groups.

**Table 3:** Genetic susceptibility factors for AIP and clinical implications.

Gene	Polymorphism/genotype	Implication	Population
Human leukocyte antigen (HLA)	DRB1*0405, DQB1*0401	Increased disease susceptibility	Japanese
	Non-aspartic acids at DQβ1 57	Increased relapse risk	Korean
Cytotoxic T lymphocyte-antigen (CTLA-4)	+49A	Increased disease susceptibility	Chinese
	+6230G/G	Increased disease susceptibility	Japanese
	+6230A	Decreased disease susceptibility	Japanese
	+49A/A, +6230A/A	Increased relapse risk	Japanese
Tumour necrosis factor-α (TNF-α)	-893A	Other organ involvement	Chinese
Fc receptor-like 3 (FCRL3)	-110A/A	Increased disease susceptibility	Japanese

In view of the autoimmune hypothesis surrounding IgG4-related disease, a series of studies have investigated the specificity of autoantibody responses in patients with type 1 AIP leading to the identification of several autoantigens. Antibodies against lactoferrin and carbonic anhydrase (CA) II are most frequently detected in type 1 AIP (73% and 54%, respectively) [82]. Anti-CA-IV antibodies were also detected in 34% of patients [83]. Other potential autoantigens include pancreatic secretory trypsin inhibitor and trypsinogens [84,85]. However, it remains to be seen whether IgG4 is an autoantibody in type 1 AIP or is over-expressed secondary in response to an unknown primary inflammatory stimulus.

Interestingly, there is significant homology between human CA-II and alpha-CA of *Helicobacter pylori *(*H. pylori*) [86]. More recently, it was found that 94% of patients with type 1 AIP had antibodies against plasminogen-binding protein of *H. pylori *[87]. The amino acid sequence of this protein exhibits homology with that of the ubiquitin-protein ligase E3 component n-recognin 2 which is expressed in pancreatic acinar cells. These two studies raise the possibility that *H. pylori *infection might trigger type 1 AIP in genetically predisposed subjects via molecular mimicry [86,87]. However, the latter study has reported elevated IgG4 in only 53% of the patients making it impossible to accurately estimate how many of these were type 1 AIP [87].

A T-helper (Th) 1 immune response has been considered to be predominant in type 1 AIP [82]. However, recent studies provided data in support of a Th2-predominant immune response in IgG4-related disease [88-92]. In addition, the number of regulatory T-cells (Tregs) is characteristically increased in tissue-resident lymphocyte populations and whole blood of patients with type 1 AIP. The number of CD4^+^CD25^high ^Tregs in blood was found significantly higher in patients with type 1 AIP than in patients with chronic pancreatitis and was correlated with the level of serum IgG4 [93]. In contrast, the number of naïve Tregs was significantly decreased. This has led to the assumption that hypo-reactivity of naïve Tregs may be involved in the development of IgG4-related disease, whereas a hyper-reactivity of CD4^+^CD25^high ^Tregs could reflect IgG4-related disease progression [93]. Given that IL-10 has a major role in directing B cells to produce IgG4, IL-10 produced from Th2 cells and Tregs may be involved in hyper-IgG4 in type 1 AIP [94].

Immune complex deposition is identifiable in the basement membrane of pancreatic ducts and acini [95,96]. These complexes consist of predominantly IgG4 and complement C3, with minor components of other IgG subclasses [96,97]. Given that IgG4 cannot form large immune complexes because of Fab-arm exchange, it remains to be clarified what is the role of IgG4 in immune complex formation, and how these depositions are involved in the pathogenesis of type 1 AIP.

## Conclusion

Type 1 AIP is a pancreatic manifestation of IgG4-related disease. Serological, imaging, and histological examination are necessary for the diagnosis. Th2-dominant immunity and activated Tregs are supposed to be important for the immunopathogensis of the disease. Unique biological characteristics of IgG4 have been identified, but the exact role of IgG4 and the relevance of these targets in the induction of the disease remain to be clarified.

## Abbreviations

AIP: autoimmune pancreatitis; ANA: antinuclear antibody; CA: carbonic anhydrase; CT: computed tomography; ERCP: endoscopic retrograde cholangiopancreatography; FDG-PET: F-18 fluorodeoxyglucose positron emission tomography; GEL: granulocytic epithelial lesion; H. Pylori: Helicobacter pylori; LPSP: lymphoplasmacytic sclerosing pancreatitis; MRCP: magnetic resonance cholangiopancreatography; MRI: magnetic resonance imaging; Th: T-helper; Treg: regulatory T-cells; US: ultrasonography.

## Competing interests

The authors declare that they have no competing interests.

## Authors' contributions

YZ and SK wrote the manuscript. All authors exchanged idea and approved the final version.
